# The Impact of Penicillin Skin Testing on Aztreonam Stewardship and Cost Savings in Immunocompromised Cancer Patients

**DOI:** 10.1093/ofid/ofz371

**Published:** 2019-08-23

**Authors:** Farnaz Foolad, Sheila Berlin, Candice White, Emma Dishner, Ying Jiang, Mahnaz Taremi

**Affiliations:** 1 Division of Pharmacy, The University of Texas MD Anderson Cancer Center, Houston; 2 Department of Infectious Diseases, Infection Control, and Employee Health, The University of Texas MD Anderson Cancer Center, Houston

**Keywords:** allergy testing, antimicrobial stewardship, aztreonam, cost savings, penicillin allergy

## Abstract

**Objective:**

Reported penicillin allergies result in alternative antimicrobial use and are associated with worse outcomes and increased costs. Penicillin skin testing (PST) has recently been shown to be safe and effective in immunocompromised cancer patients, yet its impact on antimicrobial costs and aztreonam utilization has not been evaluated in this population.

**Method:**

From September 2017 to January 2018, we screened all admitted patients receiving aztreonam. Those with a self-reported history of possible immunoglobulin E (IgE)-mediated reaction to penicillin were eligible for PST with oral challenge.

**Results:**

A total of 129 patients were screened, and 49 patients were included and underwent testing. Sixteen patients (33%) had hematologic malignancies and 33 patients (67%) had solid tumors. After PST with oral challenge, 46 patients (94%) tested negative, 1 patient tested positive on oral challenge, and 2 patients had indeterminate results. The median time from admission to testing was 2 days (interquartile range, 1–4). After testing negative, 33 patients (72%) were switched to beta-lactam therapy, which resulted in a total of 390 days of beta-lactam therapy. For identical therapy durations, the direct total antibiotic cost was $15 138.89 for beta-lactams versus $78 331.50 for aztreonam, resulting in $63 192.61 in projected savings. A significant reduction in median days of aztreonam therapy per 1000 patient days (10.0 vs 8.0; *P* = .005) was found during the intervention period.

**Conclusions:**

Use of PST in immunocompromised cancer patients receiving aztreonam resulted in improved aztreonam stewardship and significant cost savings. Our study demonstrates that PST with oral challenge should be considered in all cancer patients with reported penicillin allergies.

## Teaser

Among immunocompromised cancer patients with reported penicillin allergies, 94% tested negative on skin allergy testing with oral challenge; of these, 72% were transitioned to beta-lactam therapy, leading to significant cost savings and a reduction in institutional aztreonam use.

## INTRODUCTION

The misuse and overuse of antimicrobial agents is a serious public health issue. As a result, medical centers have established antimicrobial stewardship programs (ASPs) that stress the appropriate use of antimicrobials to reduce microbial resistance, reduce health care costs, and improve patient outcomes. Attempting to clarify old or inaccurate medication allergies, such as a penicillin allergy in a patient’s medical history, is an important feature of antimicrobial stewardship. This has led to increased focus on reported medication allergies and their relationship to suboptimal patient outcomes [1–3]. For example, hospitalized patients with penicillin allergy labels have increased healthcare costs and worse outcomes than patients without allergy labels due to avoidance of beta-lactam usage [4–6].

Reported penicillin allergies are not always accurate. Although approximately 10% of the population self-report allergies to penicillin agents, only 1% have a true penicillin allergy as determined by penicillin skin testing (PST) [7]. Thus, penicillin skin testing is emerging as an important and cost-effective component of antimicrobial stewardship. In fact, the 2016 IDSA Guidelines for Implementing an Antibiotic Stewardship Program recommend that ASPs implement allergy assessments for patients with a documented penicillin allergy [8].

Recent reports highlight the utility of PST in patients with immunoglobulin E (IgE)-mediated type 1 allergic reactions, yet immunocompromised patients frequently have been left out of allergy testing efforts because of fears of severe adverse reactions and decreased testing validity [9]. This led us to evaluate the safety, efficacy, and clinical impact of PST with oral challenge in cancer patients. We found that the utilization of PST with oral challenge in immunocompromised cancer patients, including profoundly neutropenic leukemia patients, is safe and effective for ruling out IgE-mediated penicillin allergy in this patient population. Ninety-five percent of patients who underwent PST with oral challenge in our study tested negative for penicillin allergy. The majority of these patients subsequently were changed to penicillin-based therapy with no documented adverse events [10].

Optimizing antimicrobial use is important particularly for cancer patients, because their immunocompromised status makes them vulnerable to infections and infectious complications that often require frequent antimicrobial treatments [11]. Common causes of infections in these patients include febrile neutropenia, bacteremia, and pneumonia, for which beta-lactam antimicrobials are first-line treatments [11]. Accordingly, a history of penicillin allergies significantly affects therapeutic management, because it necessitates the avoidance of penicillin agents. Often, cephalosporin and carbapenem agents are avoided as well despite data suggesting cross-reactivity rates are low [12]. A recent study by Huang et al found that among hospitalized patients with hematologic malignancies, those with reported beta-lactam allergies (of which a penicillin allergy is most common) had increased 30- and 180-day mortality rates, readmission rates, lengths of stay, and hospital charges compared to patients without beta-lactam allergies [6]. Patients with reported penicillin allergies frequently receive alternative antimicrobial agents, such as vancomycin, fluoroquinolones, and aztreonam, which are associated with increased toxicities [5, 13, 14]. In addition, aztreonam is significantly more expensive than piperacillin/tazobactam and cefepime [15–17], and because organisms, particularly *Pseudomonas aeruginosa*, show increased resistance to aztreonam, the agent is less optimal for use in this high-risk population.

Therefore, the objective of this quality improvement initiative was to improve aztreonam stewardship in cancer patients with reported penicillin allergies through PST followed by oral challenge with amoxicillin. We also assessed the impact of allergy testing on antimicrobial cost savings and institutional aztreonam utilization.

## METHODS

This study was conducted at The University of Texas MD Anderson Cancer Center, a comprehensive cancer center in Houston, Texas, and was approved by the Institutional Quality Improvement Assessment Board. The dedicated PST team consisted of an infectious diseases (ID) physician, ID fellow, ID pharmacist, and 2 advanced-practice providers (APPs). The ID pharmacist assisted with patient allergy screening and actual skin testing was conducted by the attending physician, fellow, or APPs. A report was created in the institutional electronic medical record (EMR) to capture all patients with active orders for aztreonam. All admitted patients with a reported penicillin allergy who were receiving aztreonam were screened for eligibility on weekdays (Monday–Friday) from September 2017 to January 2018. Patients were eligible for testing if they were at least 18 years old with a history of possible an IgE-mediated type 1 reaction or an unknown reaction to a PCN agent (penicillin, amoxicillin, amoxicillin/clavulanate, piperacillin/tazobactam, dicloxacillin, nafcillin, and oxacillin). Patients were excluded if they did not have a true allergy (eg, intolerance), had a non-IgE–mediated allergic reaction (eg, Stevens-Johnson syndrome, interstitial nephritis), had an anaphylactic reaction to a PCN or related agent in the last 5 years, were ≥85 years old, were in the intensive care unit or had hemodynamic instability, or were receiving an antihistamine agent that could not be discontinued. Patients also were excluded if they were receiving aztreonam as a targeted therapy for infection with a multidrug-resistant organism (such as a metallo-beta-lactamase-producing, carbapenem-resistant organism) and no other therapy options were available.

A thorough allergy history was performed and verbal consent was obtained prior to PST. Testing was conducted within 48 hours of screening on weekdays (Monday–Friday, excluding holidays). All patients had skin testing to the major determinant benzylpenicilloyl polylysine, minor determinant penicillin G potassium (10 000 units/ml), histamine positive control, and saline negative control as previously described [18]. Skin prick testing was considered positive if the wheal diameter was ≥3 mm larger than that of the negative control in the presence of a positive histamine control wheal of ≥5 mm. Intradermal skin testing was considered positive if there was an increase in size from the original wheal by ≥3 mm. All patients with negative prick and intradermal skin testing were challenged with oral amoxicillin 250 mg and were monitored for 60 minutes for any signs of hypersensitivity. If no reaction occurred, the penicillin allergy label was removed from the EMR. The primary oncology team was notified of the test results and encouraged to change therapy to an appropriate penicillin-based agent, although exact treatment recommendations were not provided. A procedure note with the test result was placed in the EMR. All patients were given a pocket card with the result of the allergy test and encouraged to share the results with any healthcare providers outside of the institution.

In addition to allergy history, the following information was collected for each enrolled patient: demographic data including age and gender, underlying malignancy, primary admitting service, admission and discharge dates, diagnoses, length of hospitalization, indication for aztreonam therapy, history of infection or colonization with drug-resistant organisms, microbiological cultures, and name and duration of therapy for all antibiotics given during admission and upon discharge.

The cost savings associated with change of therapy to beta-lactam agents was calculated based on the wholesale acquisition cost of antimicrobial agents with the assumption of a full daily dose per day and normal renal function [17]. Utilization of aztreonam at our institution prior to the aztreonam-targeted PST initiative (September 2016–August 2017) and during the aztreonam-targeted PST initiative (September 2017–January 2018) was analyzed and reported in days of therapy (DOT) per 1000 patient days. There were no other efforts targeting aztreonam use during the time of this study and the agent is not restricted at our institution.

### Statistical Analysis

Descriptive statistics were used to summarize the data. Continuous variables were presented as the median and interquartile range (IQR) or range. Categorical variables were presented as the frequency and percentage. The Wilcoxon rank sum test was used to compare aztreonam DOT before and after intervention. A *P* value of ≤.05 was considered statistically significant. Statistical analyses were performed using SAS version 9.3 (SAS Institute, Inc., Cary, NC).

## RESULTS

### Screening and Patient Demographics

Over the study period, 129 hospitalized patients with an active order for aztreonam were screened for inclusion. Of these patients, 49 met the inclusion criteria, provided verbal consent, and underwent PST. The most common reasons for not testing were patient refusal (19%) and hemodynamic instability (13%) (Supplementary Table 1). The patient demographics are shown in Table 1. Of the 49 patients tested, 24 (49%) were male, the median age at testing was 68 years (range, 23–84) and the majority (67%) had an underlying solid tumor malignancy. Hives (36%) and anaphylaxis (18%) were the most commonly reported reactions to penicillins. The median absolute neutrophil count (ANC) was 2.4 k/μL in patients with hematologic malignancies and 4.8 k/μL in those with solid tumors.

**Table 1.** Demographics and Characteristics of the Patients Who Underwent Penicillin Skin Testing

Characteristics	N = 49 N (%)
Age (y), median (range)	68 (23–84)
Sex, Male	24 (49)
Length of stay, median days (range)	5 (2–38)
Type of cancer	
Leukemia	4 (8)
Lymphoma/myeloma	12 (24)
Solid tumor	33 (67)
Labs, hematologic malignancies (n = 16), median (k/μL) (range)	
WBC	3.8 (0.40–72.2)
ANC	2.4 (0.01–76.3)
ALC	0.7 (0.02–65.0)
Platelets	112.5 (21.0–262.0)
Labs, solid tumors (n = 33), median (k/μL) (range)	
WBC	6.8 (0.90–19.0)
ANC	4.8 (0.18–16.1)
ALC	0.6 (0.07–26.1)
Platelets	169.0 (24.0–423.0)
Antibiotic allergy	
Penicillin (IV or oral)	47 (96)
Amoxicillin or amoxicillin/clavulanate	2 (4)
Piperacillin/tazobactam	1 (2)
Reported reaction^a^	
Childhood reaction	1 (2)
Hives	18 (36)
Itchiness	2 (4)
Skin rash (not specified as hives)	6 (12)
Swelling or angioedema	5 (10)
Syncope	1 (2)
Unknown	1 (2)
Duration between admission and PST in days, median (IQR)	2 (1–4)

Data are presented as number (%) of patients unless otherwise indicated.

Abbreviations: ALC, absolute lymphocyte count; ANC, absolute neutrophil count; IQR, interquartile range; IV, intravenous; PST, penicillin skin testing; WBC, white blood cells.

^a^ Patients may report more than 1 reaction.

The most common indication for antibiotic use was pulmonary infection (29%), followed by nonneutropenic fever of unknown origin (20%), and urinary tract infection (14%) (Table 2**).** All patients tested were receiving aztreonam therapy due to a reported penicillin allergy. There were no patients tested that were receiving aztreonam due to a history of, or current infection with, an organism with a resistance pattern necessitating aztreonam use. Thirty-five patients (71%) were on concomitant broad-spectrum gram-positive coverage (ie, vancomycin, daptomycin, or linezolid), and 5 patients (10%) received concurrent fluoroquinolone therapy.

**Table 2.** Indications for Antibiotic Therapy and Culture Findings in Patients Who Underwent Penicillin Skin Testing

Variable	N = 49 N (%)
Admitting diagnosis	
Bacteremia	4 (8)
Bone and joint infection	1 (2)
Sepsis	1 (2)
Intraabdominal infection	2 (4)
Neutropenic fever of unknown origin	5 (10)
Nonneutropenic fever of unknown origin	10 (20)
Oral infection	1 (2)
Pulmonary infection	14 (29)
Skin and soft tissue infection	4 (8)
Urinary tract infection	7 (14)
Positive cultures during admission^a^	14 (29)
Blood	8
Respiratory	1
Urine	7
Other	2
Gram-negative organisms identified during hospitalization	
*Citrobacter* species	1
*Escherichia coli*	5
*Klebsiella* species	2
*Pseudomonas aeruginosa*	4
*Stenotrophomonas maltophilia*	1
*Serratia* species	1
Polymicrobial	2
Gram-positive organism identified during hospitalization	
Coagulase-negative *Staphylococci*	2
*Enterococcus faecalis*	2
Enterococcus species	1
*Staphylococcus aureus*	2
*Staphylococcus lugdunensis*	1
*Streptococcus* species	1
Total days of aztreonam, median (interquartile range)	2 (2–3)
Additional antimicrobials ordered with aztreonam	42 (86)
Azithromycin	1 (2)
Amikacin	1 (2)
Bactrim	1 (2)
Ciprofloxacin	3 (7)
Daptomycin	2 (5)
Doxycycline	3 (7)
Gentamicin	1 (2)
Levofloxacin	2 (5)
Linezolid	6 (14)
Metronidazole	9 (21)
Vancomycin	27 (64)

^a^ Patients may have had more than 1 positive culture.

### PST Results

**Figure 1.**
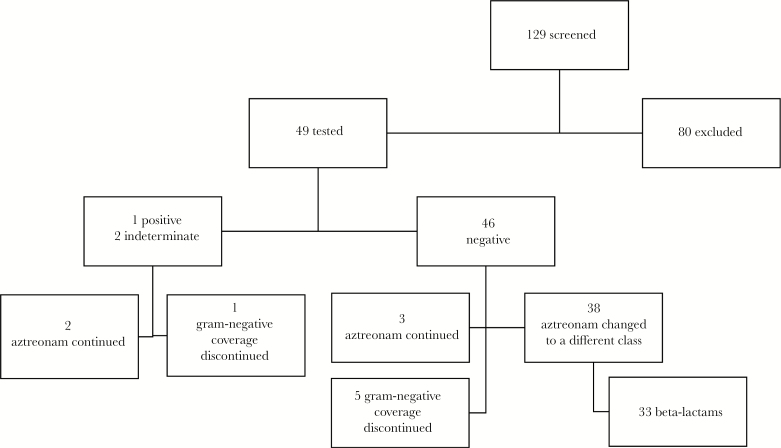
Results of Penicillin Skin Testing With Oral Challenge and Subsequent Changes in Aztreonam Therapy

The median time from admission to PST was 2 days (IQR, 1–4) (Table 2). Of the 49 patients who underwent PST, 46 (94%) were negative on both PST and oral challenge (Figure 1). One patient (2%) had a negative PST but developed a delayed maculopapular cutaneous reaction approximately 3 hours after the oral challenge. This patient was continued on aztreonam for 5 days and then was discharged on oral levofloxacin for possible pneumonia. The PST result was indeterminate in 2 patients (4%) due to negative histamine control responses, and allergy could not be ruled out. Neither of these 2 patients were neutropenic (ANCs 2.4 and 2.7 k/μL) or lymphopenic (ALC 0.9 and 0.8 k/μL) at the time of attempted test. Of these, 1 had aztreonam discontinued the day after PST due to low suspicion for infection, and the other was continued on aztreonam for 17 days of therapy for foot cellulitis in combination with gram-positive coverage.

### Antimicrobial Changes in Patients Negative on PST and Oral Challenge

Ninety-eight percent (46 out of 47) of the patients who completed PST and oral challenge had negative results. Of these patients, 5 had aztreonam discontinued within 24 hours of testing due to a low suspicion for a gram-negative infectious process (Figure 1). Of the remaining 41 patients, 3 patients were continued on aztreonam due to provider preference despite a negative testing result; each received 1 or more additional concomitant antimicrobial therapies (vancomycin, linezolid, doxycycline, ciprofloxacin, or levofloxacin) (Supplementary Table 2). Five patients were switched from aztreonam to an alternative, non-beta-lactam agent. The remaining 33 patients were switched to a beta-lactam agent: 19 patients (41%) received penicillins, 16 patients (35%) received cephalosporins, and 4 (9%) patients received carbapenems. Five of these patients received more than 1 class of beta-lactam (4 patients received 2, 1 patient received 3), resulting in a total of 39 beta-lactam exposures for the initial infectious episode (Table 3). The most common beta-lactams prescribed after PST were cefepime (12 patients) and amoxicillin/clavulanate (11 patients). Summaries of other antimicrobial exposures are provided in Supplementary Tables 2 and 3. There were no documented reactions due to use of beta-lactams after a negative result on PST and oral challenge.

**Table 3.** Beta-Lactam Antibiotics Prescribed on an Inpatient and Outpatient Basis After Negative Penicillin Skin Testing

Antibiotic^a^	Patients (N = 33)	Days of Beta-Lactam use		
	N (%)	Median^b^	Range^c^	Sum
Amoxicillin	3 (8)	6	6–8	20
Amoxicillin/clavulanate	11 (28)	9	6–14	101
Ampicillin/sulbactam	4 (10)	10	3–45	68
Cefazolin	1 (3)	13	—	13
Cefepime	12 (30)	3	2–14	59
Cefpodoxime	4 (10)	6	5–9	25
Ceftriaxone	2 (5)	22	1–42	43
Ertapenem	1 (3)	5	—	5
Imipenem	1 (3)	2	—	2
Meropenem	2 (5)	5	1–8	9
Nafcillin	1 (3)	27	—	27
Piperacillin/tazobactam	4 (10)	5	2–7	18
Beta-lactam exposure by class				
Penicillins	19 (48)	10	6–45	234
Cephalosporins	16 (40)	5	2–42	140
Carbapenems	4 (10)	4	1–8	16
Total				390

^a^Patients may have received more than 1 type of beta-lactam antibiotic.

^b^The number given is the actual number of days of beta-lactam use if only 1 patient received the drug in question.

^c^No range is given if only 1 patient received the drug in question.

### Cost Savings Due to Switch to Beta-Lactam Therapy

In total, 33 patients (72%) who tested negative for penicillin allergy (PST and oral challenge) were transitioned to a beta-lactam and received a total of 390 days of therapy (234 days of penicillins, 140 days of cephalosporins, and 16 days of carbapenems). The median duration of aztreonam therapy was 2 days (IQR, 2–3). For patients switched to a beta-lactam, total cost of antibiotic therapy was $15 138.89 based on wholesale acquisition costs. For the same duration of therapy, the estimated cost of aztreonam therapy would be $78 331.50. Switching from aztreonam therapy to beta-lactam therapy for treatment of patients’ initial infectious processes resulted in a cost savings of $63 192.61 ($1914.93 per patient).

### Aztreonam Utilization

**Figure 2.**
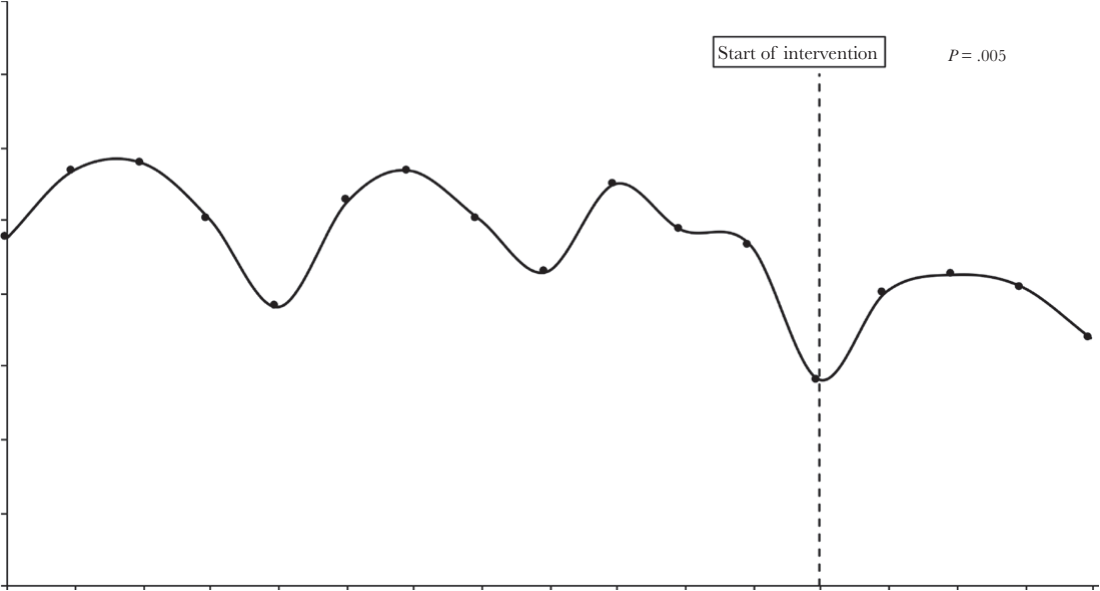
Days of Therapy per 1000 Patient Days Present for Aztreonam in the Preintervention Versus Intervention Periods

Compared to the preaztreonam-targeted intervention period (September 2016–August 2017), the aztreonam DOT per 1000 patient days during the aztreonam-targeted PST intervention period (September 2017–January 2018) significantly decreased from a median of 10.0 (IQR, 9.4–11.2) to 8.0 (IQR, 6.8–8.2) (*P* = .005) (Figure 2).

DOT indicates days of therapy.

## DISCUSSION

Self-reported penicillin allergies remain a significant patient care issue and lead to increased healthcare costs and suboptimal clinical outcomes [5, 6]. Although efforts to clarify allergies and conduct allergy testing have increased in recent years, immunocompromised patients often have been excluded from these efforts. Our recently published study demonstrated the safety and efficacy of PST in immunocompromised cancer patients, including those receiving chemotherapy with profound neutropenia and thrombocytopenia [10]. In the current study, we demonstrate the feasibility of PST with oral challenge for immunocompromised cancer patients on aztreonam therapy. These patients are among those at highest need for immediate allergy clarification. In our study, PST with oral challenge enabled the majority of immunocompromised cancer patients (72%) to be switched from aztreonam therapy to the preferred beta-lactam therapy. This resulted in significant antimicrobial cost savings and a significant reduction in institutional aztreonam drug utilization.

Various methods of implementing PST services have been described in the literature [19–22]. A successful PST program requires dedicated time and resources and must be customized to institutional needs and goals. Our institution, a 600-bed comprehensive cancer center, serves a large number of immunocompromised cancer patients, many of who are frequently hospitalized for infections. It is not feasible to test all admitted patients who self-report penicillin allergies; thus, a more targeted approach was necessary. Previous studies have demonstrated the feasibility of targeting patients who received second-line antibiotics for PST evaluation, although none have evaluated this approach in an immunocompromised population [22–24]. Our inpatient antibiogram data revealed decreased susceptibility of *Pseudomonas aeruginosa* isolates to aztreonam (78%) compared to other beta-lactams, including meropenem (88%), cefepime (87%), ceftazidime (90%), and piperacillin-tazobactam (85%). As a result, we focused our initiative on patients being actively treated with aztreonam whom we believed would benefit most from allergy delabeling. This targeted approach successfully limited aztreonam use to a median of 2 days. Forty-six patients were negative on PST and oral challenge, and 33 of these patients were immediately changed to beta-lactam therapy, which is the preferred therapy for the majority of infections treated in this patient population. Although we only evaluated antimicrobial usage during the initial infectious episode, patients who tested negative had their penicillin allergies fully removed from the EMR. Removing the penicillin allergy from the EMR will optimize future antimicrobial therapy for these patients during the remainder of their cancer care.

In addition to optimizing antimicrobial therapy and allergy documentation, our aztreonam-directed PST service resulted in significant antimicrobial cost savings. The switch from aztreonam to beta-lactam therapy resulted in a cost savings of $1914.93 per patient for the initial infectious episode. Blumenthal et al estimated the cost of a penicillin allergy evaluation to be $220 per patient, a relatively modest cost when compared to the antimicrobial cost savings achieved with our intervention [25]. Cancer patients, particularly those with hematologic malignancies, are frequently admitted for infectious complications, and, although subsequent cost savings were not directly measured in our study, it is likely that removing penicillin allergies from the EMR would result in continued cost savings throughout the course of cancer care.

Lastly, aztreonam-targeted PST decreased the utilization of aztreonam at our institution. Aztreonam is not restricted at our institution; therefore, it is often the preferred agent for those with reported PCN allergies. The median DOT per 1000 patient days was significantly reduced by 20%. Similar findings have been published in studies of immunocompetent patients [24]. Although aztreonam utilization was reduced at our institution, its use still persisted (albeit at a lower level) during the study period. Although there are certain indications for which aztreonam is necessary (ie, infection with multidrug-resistant organisms only susceptible to aztreonam), most orders for aztreonam in our patient population were driven by self-reported PCN allergies, demonstrating additional room for improvement. During our study period, 15 screened patients refused testing, the majority of who cited the burden of their cancer diagnosis and related therapies as the main reason for refusal. Identifying ways to capture such patients for allergy testing, whether through enhanced patient education or the availability of PST in outpatient clinics prior to initiation of chemotherapy, might help to further decrease overall aztreonam use. Moreover, recent reports highlighting the feasibility of a modified allergy assessment using only oral challenge in low-risk patients may be appealing to those who wish to avoid the full skin-testing procedure [26].

Strengths of our study include its targeting of patients with a high need for allergy clarification, its use of a multidisciplinary team for patient assessment and testing, and its inclusion of a full spectrum of oncologic patients, including those with hematologic malignancies. Our study was not without limitations, however. Aztreonam screening was only performed during weekdays, potentially prolonging aztreonam therapy on days when PST services were not available (ie, Saturday and Sunday). In addition, although primary teams were notified of the PST results, the PST team did not directly make therapeutic recommendations. Patient-specific therapeutic recommendations from the PST team and ID consult services may enhance the immediate optimization of antimicrobial therapy after PST. Moreover, 3 patients who tested negative on PST and oral challenge continued aztreonam due to physician preference. This suggests that increased efforts are necessary to educate healthcare providers on the PST procedure and the relevance of the results.

In summary, aztreonam-targeted PST with oral challenge resulted in significant optimization of antimicrobial therapy in a spectrum of immunocompromised cancer patients. The majority of PST negative patients were transitioned to beta-lactam therapy. This effort resulted in significant cost savings and a reduction in aztreonam use, which supports institutional antimicrobial stewardship efforts. These findings further support the use of PST in immunocompromised patients and demonstrate that there are both clinical and financial benefits to performing PST for cancer patients with reported penicillin allergies.

## Supplementary Data

Supplementary materials are available at *Open Forum Infectious Diseases* online. Consisting of data provided by the authors to benefit the reader, the posted materials are not copyedited and are the sole responsibility of the authors, so questions or comments should be addressed to the corresponding author.

ofz371_suppl_supplementary_material
