# Supplementary material for: The Impact of Penicillin Skin Testing on Aztreonam Stewardship and Cost Savings in Immunocompromised Cancer Patients
Source: Open Forum Infect Dis. 2019 Aug 23;6(10):ofz371. doi: 10.1093/ofid/ofz371 (PMC6767966; doi:10.1093/ofid/ofz371)
Supplement: ofz371_suppl_supplementary_material [file ofz371_suppl_supplementary_material.docx]

**Supplementary Table 1: Summary of reasons for exclusion after screening for PST**

| Reason for exclusion | N=80  N (%) |  |
| --- | --- | --- |
| Patient refused | 15 (19) |  |
| Hemodynamically unstable | 10 (13) |  |
| Age ≥ 85 | 9 (11) |  |
| In the ICU | 7 (9) |  |
| Recent allergic reaction* | 5 (6) |  |
| Anaphylaxis from PCN or related agent in last 5 years | 4 (5) |  |
| Non-IgE-mediated reaction to PCN | 3 (4) |  |
| Allergy updated, corrected (testing not done) | 3 (4) |  |
| On antihistamines unable to be discontinued | 2 (3) |  |
| Discharged prior to eligibility | 4 (5) |  |
| Other | 20 (25) | Cephalosporin allergy (not penicillin allergy): 6  Previous positive PST test within prior year: 3  Not safe to test: 2 (1, recent seizure; 1, airway tumor)  SCT patient: 2  Primary team refused: 1  Planned hospice: 1  Altered mental status (could not consent): 1  No PCN allergy on patient interview: 1  Aztreonam being used for MDR infection: 1 |

ICU, intensive care unit; IgE, immunoglobulin E; MDR, multidrug-resistant; PCN, penicillin; PST, penicillin skin testing; SCT, stem cell transplant.

*Patients that had an inpatient, physician observed reaction to a penicillin agent at MD Anderson within the last 12 months

**Supplemental Table 2: Antibiotics prescribed with aztreonam in patients continued on aztreonam despite negative PST and oral challenge results**

|  | |
| --- | --- |
| Antibiotic | **Patients (N=3)^a^**  **N (%)** |
| Ciprofloxacin | 1 (33) |
| Doxycycline | 1 (33) |
| Levofloxacin | 2 (67) |
| Linezolid | 1 (33) |
| Vancomycin | 1 (33) |

^a^All 3 patients received more than one antibiotic.

**Supplemental Table 3: Concurrent antibiotics given to patients who were switched from aztreonam therapy^a^**

| Antibiotic | Patients (N=38)  N (%) |
| --- | --- |
| Azithromycin | 1 (3) |
| Ciprofloxacin | 4 (11) |
| Daptomycin | 2 (5) |
| Levofloxacin | 8 (21) |
| Linezolid | 3 (8) |
| Metronidazole | 3 (8) |
| Minocycline | 1 (3) |
| Nitrofurantoin | 2 (5) |
| Tigecycline | 1 (3) |
| Trimethoprim/sulfamethoxazole | 1 (3) |
| Vancomycin | 3 (8) |

^a^Beta-lactam antibiotics given to these patients are listed in Table 5.
